# Association between composite dietary antioxidant index and cognitive function impairment among the US older adults: a cross-sectional study based on the NHANES 2011–2014

**DOI:** 10.3389/fnut.2024.1471981

**Published:** 2024-11-22

**Authors:** Cong Zhao, Meng Pu, Chengji Wu, Jiaqi Ding, Jun Guo, Guangyun Zhang

**Affiliations:** ^1^Department of Neurology, Air Force Medical Center of PLA, Beijing, China; ^2^Department of Hepatobiliary Surgery, Air Force Medical Center of PLA, Beijing, China; ^3^Department of Neurology, Tangdu Hospital, Air Force Medical University, Xi’an, China

**Keywords:** composite dietary antioxidant index, cognitive function impairment, Alzheimer’s disease, cross-sectional study, NHANES

## Abstract

**Background:**

Cognitive function impairment (CFI) and the Composite Dietary Antioxidant Index (CDAI) were investigated in this study.

**Methods:**

Participants from the 2011–2014 cycles of the National Health and Nutrition Examination Survey were chosen to assess cognitive function using the Consortium to Establish a Registry for Alzheimer’s Disease Word Learning Test, the Animal Fluency Test, and the Digit Symbol Substitution Test. Participants scored below the 25% percentile of any of the three tests were defined as having cognitive function impairment. 24-h recalls of diet were collected to calculate CDAI.

**Results:**

2,424 participants were included. The fully adjusted multivariate logistic regression model showed an increase of one CDAI unit reduced CFI risk by 5% (95% CI: 0.92 ~ 0.98, *p* = 0.004). When comparing individuals with the lowest CDAI in the first quartile (<−2.42), the adjusted odds ratio for CDAI and CFI were 0.81 (95% CI: 0.61 ~ 1.06, *p* = 0.125) in the second quartile, 0.69 (95% CI: 0.51 ~ 0.92, *p* = 0.012) in the third quartile, and 0.59 (95% CI: 0.43 ~ 0.82, *p* = 0.002) in the fourth quartile, respectively. Restricted cubic spline analysis revealed a steady negative linear correlation between CDAI and CFI, with a *p*-value for non-linearity of 0.122. Subgroup analysis did not reveal any significant interactions based on age, education level, family income, history of diabetes, hypertension, stroke, and depression.

**Conclusion:**

CDAI was inversely associated with CFI in a large representative American population. Further longitudinal studies are needed for causal inference.

## 1 Introduction

Age-related cognitive decline has emerged as a significant health challenge in an era of extended life expectancy. Approximately one-third of Americans aged 65 and older experience mild cognitive impairment (MCI) or dementia, which significantly impacts personal relationships, quality of life, and healthcare systems (1, 2). In fact, the cost of individuals with low cognitive abilities was estimated to reach 345 billion dollars in the United States alone in 2023 (2). While effective treatments for dementia remain elusive, understanding the factors contributing to cognitive decline is crucial for prevention and improved quality of life.

Emerging evidence indicates that a decrease in cognitive function is linked to oxidative stress (3, 4), which is caused by an imbalance between antioxidants and reactive oxygen species (ROS) and reactive nitrogen species (RNS). The brain could be greatly harmed by oxidative stress, which has been proven to play a major role in the pathogenesis of Alzheimer’s disease (AD) (5, 6). Therefore, effective prevention of oxidative stress has become a key focus. Previous studies has suggested that a diet rich in antioxidants could have a beneficial effect on cognitive function by slowing age-related changes in neurons and offering defense against the impact of oxidative stress (7–9). A study conducted in Singapore revealed that a greater overall antioxidant capacity in the diet was associated with a lower likelihood of cognitive decline in Chinese individuals (10). Therefore, modifying dietary patterns may help mitigate cognitive decline.

Numerous studies have investigated how individual antioxidant nutrients affect cognitive abilities, but assessing the combined effect of multiple antioxidant nutrients is still challenging. The composite dietary antioxidant index (CDAI) has been suggested as a dependable tool for assessing the combined antioxidant content of a person’s diet (11). The index provides a thorough summary of the dietary antioxidants, combining six essential nutrients including selenium, zinc, carotenoids, vitamin A, vitamin C and vitamin E, into a single summary score. Recent studies has established associations between CDAI and various health conditions, including hypertension (12, 13), stroke (14, 15), depression (16, 17), coronary heart disease (18), cancer (19), bone mineral density (20, 21) and hyperlipidemia (22). All these studies indicated that the level of CDAI were inversely associated with these diseases, highlighting the potential protective role of antioxidants. Yet, the possible link between CDAI and cognitive function remains underexplored. In this cross-sectional study, we investigated the association between CDAI and cognitive decline using data from the National Health and Nutrition Examination Survey (NHANES) database.

## 2 Methods

### 2.1 Study population

NHANES, carried out by the US National Center for Health Statistics (NCHS), is a detailed health study designed to assess the health and nutritional status of adults and children in the United States Each survey cycle includes a sample of the population that represents the entire nation, involving interviews, examinations, and laboratory tests to provide a comprehensive view of Americans’ health. Before joining the study, all participants have given written consent, and the ethical approval for the NHANES has been granted by the NCHS Ethics Review Committee.

**Figure 1:**
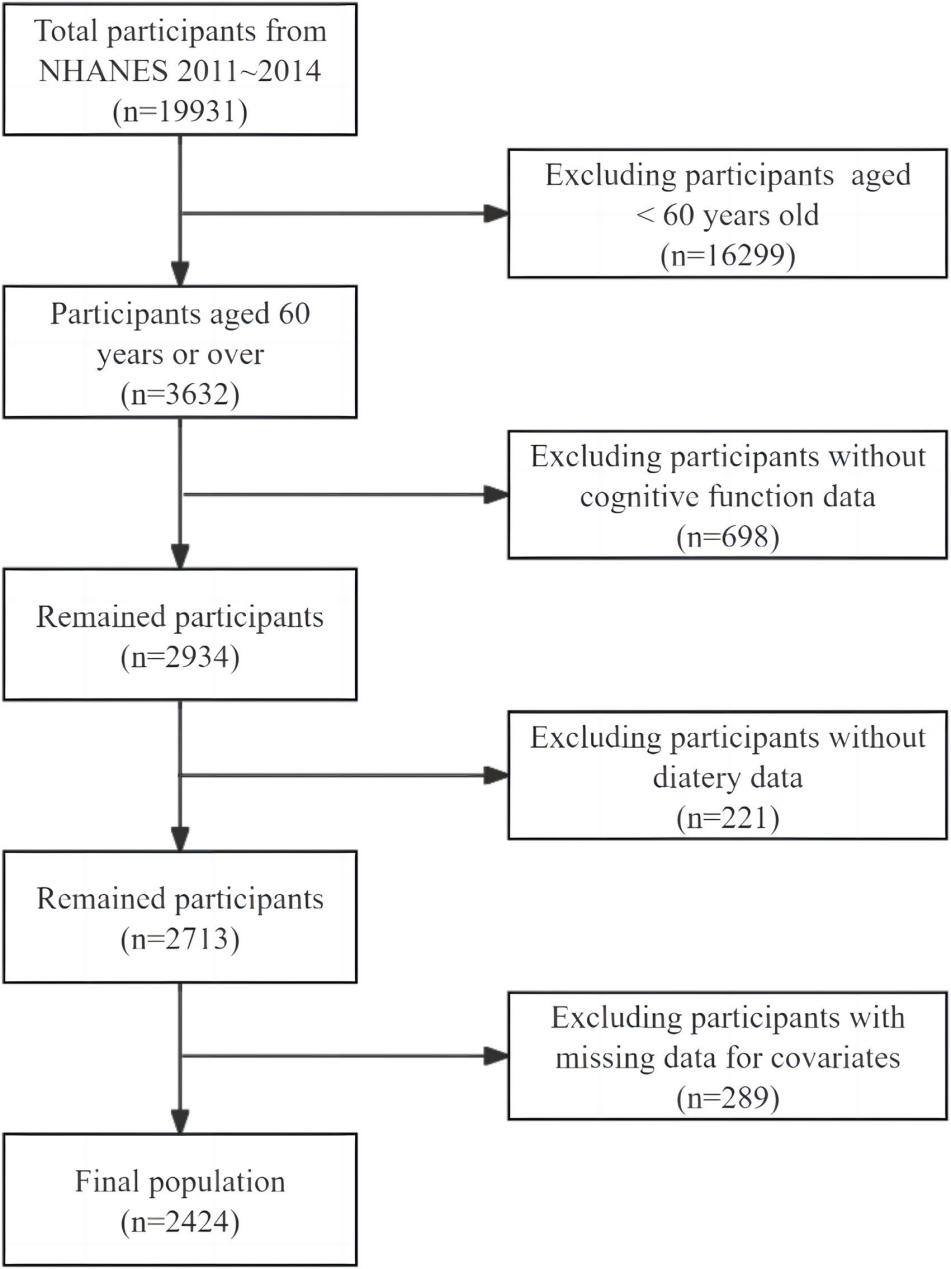
Flowchart of sample selection from the NHANES 2011–2014.

We utilized publicly accessible data from individuals recruited between 2011 and 2014, which provides results on three cognitive tests administered to individuals aged 60 years and above. Participants under the age of 60 (*n* = 16,299) and those who did not complete all cognitive assessments (*n* = 698) were excluded. Additionally, participants with missing dietary data to calculate CDAI (*n* = 221) and participants without covariates data were also excluded (*n* = 289). In total, 2,424 individuals were included in this study finally, as shown in Figure 1.

### 2.2 CDAI measurement

Thorough dietary information was gathered from NHANES participants by skilled interviewers. The interviews gathered details on the participants’ consumption of various foods, drinks, and supplements through two-day recalls that were not consecutive. The first meeting was held face-to-face at the Mobile Examination Center (MEC), with the second interview taking place over the phone, between 3 and 10 days afterwards. Daily average intakes were determined using data from the 2 days recall. We employed a modified version of CDAI (11, 23, 24). The composite score is calculated based on the daily consumption of six antioxidants: selenium, zinc, carotenoids, vitamin A, vitamin C, vitamin E. To normalize the intakes, we began by subtracting the average intake of each antioxidant and then dividing the outcome by the standard deviation (SD):


$$\mathrm{CDAI}=\overset{n=6}{\underset{i=1}{\sum }}\frac{\mathrm{individual}\;\mathrm{intake}-\mathrm{mean}}{SD}$$

### 2.3 Assessment of cognitive function

NHANES evaluated cognitive function in the MEC through three standardized tests: the Consortium to Establish a Registry for Alzheimer’s Disease (CERAD) Word Learning test, the Animal Fluency Test (AFT), and the Digit Symbol Substitution Test (DSST).

The CERAD test includes three learning trials and a delay recall trial to evaluate immediate and delayed verbal learning abilities. Participants were instructed to vocalize 10 different nouns shown on the screen during each learning trial and recall as many words as possible. The delayed test took place following completion of the AFT and DSST, typically 8–10 min after the initial learning session. During each attempt of the CERAD exam, the result varied between 0 and 10. The total score was determined by adding together the results of the three initial learning tests and delayed recall test.

The AFT evaluates executive function by requiring participants to list as many animals as possible within a minute. The scoring is based on the total count of correctly named animals.

The DSST evaluates attention and processing speed. The process was completed with the assistance of a document featuring a legend at the top, connecting numerical values to nine unique icons. Attendees were given a brief period of 2 min to pair the correct icon from the 133 boxes next to the corresponding number. The score was calculated by summing the accurate matches they made, with possible scores ranging from 0 to 133.

There is no gold standard cut-off value to identify low cognitive performance currently. According to previous studies, we used the 25th percentile as the cut-off value. Participants who scored below the cut-off values of any of the three tests were defined as having cognitive function impairment (CFI) (25, 26).

### 2.4 Covariates

We investigated various potential covariates. Demographic variables such as age, gender, race, education level, marital status, family income, and body mass index (BMI), as well as lifestyle factors like smoking status and alcohol consumption, were considered. Additionally, we examined the impact of comorbidities such as hypertension, diabetes, stroke, and depression, as well as nutrients intake including total energy, total protein, total carbohydrate and total fat on the outcome (25, 27). There were four categories used to classify race: Hispanic, non-Hispanic White, non-Hispanic Black and others. The education level was categorized into three groups: less than high school, high school and more than high school. Participants’ marital status was divided into two categories: married or living with a partner, and living alone. Family income was classified according to the poverty-to-income ratio (PIR), where a PIR less than 1.3 was labeled as low income and a PIR of 1.3 or higher was categorized as medium or high income (28). Participants’ smoking habits were classified into three groups: never (those who never smoked or have smoked less than 100 cigarettes in their lifetime), former (those who have smoked at least 100 cigarettes but are not currently smoking), and current (those who have smoked at least 100 cigarettes and are currently smoking). Those who drink a minimum of 12 alcoholic beverages annually were categorized as drinkers. History of hypertension, diabetes and stroke was defined based on self-reported physician diagnoses of these conditions. The 9-item Patient Health Questionnaire (PHQ-9) was used to assess depression. Those who scored 10 or higher were classified as having depression (29). Total intake of each nutrient was obtained through the dietary recall interview.

### 2.5 Statistical analyses

To determine the normal distribution of variables, we utilized either the histogram distribution, Q-Q plot, or the Kolmogorov–Smirnov test. Continuous variables with a normal distribution were presented as mean (SD), whereas those with skewness were reported as median (interquartile range [IQR]). Frequencies and percentages were used to present categorical variables. We used one-way ANOVA to analyze continuous variables and chi-square tests for categorical variables in order to evaluate statistical variances across the four groups. We used logistic regression to calculate the odds ratio (OR) and 95% confidence interval (CI) to assess the association between CDAI and CFI, considering CDAI as either a continuous variable or a categorical variable divided into 4 quantiles. The selection of potential covariates was guided by prior research, their significance in univariate analysis, or any notable change in the effect estimate exceeding 10%. Model 1 accounted for age, race, gender, education, marital status, and family income. Model 2 was additionally modified to account for BMI, smoking status, alcohol consumption, total energy, total protein, total carbohydrate, and total fat. Model 3 additionally accounted for concurrent health conditions (hypertension, diabetes, stroke, and depression) based on Model 2. A restricted cubic spline was used to evaluate the possible non-linear relationship between CDAI and CFI, with four knots placed at the 5th, 35th, 65th, and 95th percentiles. Additionally, a subgroup analysis was performed to explore if the correlation between CDAI and CFI was influenced by factors such as age, education level, family income, diabetes, hypertension, stroke, and depression, utilizing a likelihood ratio test. To ensure the trustworthiness of our study, individuals with very high or very low total energy intake, specifically those consuming less than 500 kcal or more than 5,000 kcal daily, were excluded for a sensitivity analysis. Linear regression were utilized to examine the relationship between CDAI and the scores of CERAD test, AFT and DSST. Statistical analyses were performed with the R Statistical Software (Version 4.2.2,^1^ The R Foundation) and Free Statistics analysis platform (Version 1.9, Beijing, China).^2^ Statistical significance was determined with a *p*-value below 0.05 on both sides.

## 3 Results

### 3.1 Baseline characteristics

In Table 1, a summary of the baseline characteristics for all 2,424 participants is shown, categorized by their CDAI quartiles. Notably, individuals in the highest CDAI quartile group are predominantly male and non-Hispanic White. They typically have a higher education level, cohabitate with a partner, have a higher family income, and maintain a lower BMI. Additionally, there were significant differences in smoking status, drinking, hypertension, diabetes, and depression across the four groups (*p* < 0.05). Crucially, the score of CERAD test, AFT and DSST vary significantly among different CDAI quartile group (*p* < 0.05), with higher cognitive scores corresponding to higher CDAI levels.

**Table 1:** Baseline characteristics of participants stratified by quartile of CDAI.

	Total (*n* = 2,424)	Q1 (<−2.42, *n* = 606)	Q2 (−2.42 ~ −0.67, *n* = 606)	Q3 (−0.67 ~ 1.62, *n* = 606)	Q4 (≥ − 1.62, *n* = 606)	*p-*value
Age group						0.084
60–69 years	1,337 (55.2)	346 (57.1)	353 (58.3)	322 (53.1)	316 (52.1)	
70–79 years	714 (29.5)	184 (30.4)	159 (26.2)	179 (29.5)	192 (31.7)	
80+ years	373 (15.4)	76 (12.5)	94 (15.5)	105 (17.3)	98 (16.2)	
Gender						< 0.001
Male	1,187 (49.0)	251 (41.4)	279 (46)	310 (51.2)	347 (57.3)	
Female	1,237 (51.0)	355 (58.6)	327 (54)	296 (48.8)	259 (42.7)	
Race						< 0.001
Hispanic	440 (18.2)	138 (22.8)	137 (22.6)	92 (15.2)	73 (12)	
Non-Hispanic White	1,219 (50.3)	241 (39.8)	276 (45.5)	328 (54.1)	374 (61.7)	
Non-Hispanic Black	565 (23.3)	186 (30.7)	149 (24.6)	128 (21.1)	102 (16.8)	
others	200 (8.3)	41 (6.8)	44 (7.3)	58 (9.6)	57 (9.4)	
Education level						< 0.001
Less than High school	565 (23.3)	205 (33.8)	151 (24.9)	121 (20)	88 (14.5)	
High school	566 (23.3)	161 (26.6)	152 (25.1)	134 (22.1)	119 (19.6)	
More than High school	1,293 (53.3)	240 (39.6)	303 (50)	351 (57.9)	399 (65.8)	
Marital status						< 0.001
Married or living with a partner	1,410 (58.2)	298 (49.2)	349 (57.6)	351 (57.9)	412 (68)	
Living alone	1,014 (41.8)	308 (50.8)	257 (42.4)	255 (42.1)	194 (32)	
Family income						< 0.001
<1.3	695 (28.7)	247 (40.8)	177 (29.2)	150 (24.8)	121 (20)	
≥1.3	1729 (71.3)	359 (59.2)	429 (70.8)	456 (75.2)	485 (80)	
BMI (kg/m^2^)	29.2 (6.5)	29.8 (7.1)	29.6 (6.3)	29.3 (6.3)	28.3 (5.9)	< 0.001
Smoking status						< 0.001
Never	1,183 (48.8)	290 (47.9)	288 (47.5)	317 (52.3)	288 (47.5)	
Former	943 (38.9)	196 (32.3)	253 (41.7)	230 (38)	264 (43.6)	
Current	298 (12.3)	120 (19.8)	65 (10.7)	59 (9.7)	54 (8.9)	
Drinking	1,683 (69.4)	372 (61.4)	417 (68.8)	439 (72.4)	455 (75.1)	< 0.001
Hypertension	1,518 (62.6)	406 (67)	386 (63.7)	368 (60.7)	358 (59.1)	0.024
Diabetes	566 (23.3)	160 (26.4)	164 (27.1)	134 (22.1)	108 (17.8)	< 0.001
Stroke	164 (6.8)	43 (7.1)	39 (6.4)	39 (6.4)	43 (7.1)	0.936
Depression	211 (8.7)	71 (11.7)	54 (8.9)	44 (7.3)	42 (6.9)	0.012
Total energy (kcal/day)	1837.0 (693.5)	1339.4 (475.2)	1744.1 (500.3)	1960.6 (621.2)	2303.9 (757.5)	< 0.001
Total protein (g/day)	73.1 (30.0)	51.5 (18.0)	68.8 (20.9)	79.1 (26.8)	93.2 (34.7)	< 0.001
Total carbohydrate (g/day)	223.3 (88.6)	168.6 (66.8)	212.9 (70.0)	236.7 (80.7)	274.8 (98.3)	< 0.001
Total fat (g/day)	70.6 (33.6)	48.4 (21.0)	67.3 (25.4)	75.7 (31.4)	90.9 (38.8)	< 0.001
Carotenoids (μg/day)	6198.0 (2892.6, 12417.8)	2744.5 (1182.1, 4934.4)	5725.0 (2908.9, 8901.0)	8146.8 (4460.5, 14241.0)	13044.2 (6227.4, 21966.1)	< 0.001
Vitamin C (mg/day)	108.4 (51.5, 208.6)	38.8 (19.0, 77.2)	85.6 (49.8, 137.2)	141.1 (88.3, 222.9)	262.1 (149.1, 601.9)	< 0.001
Vitamin E (mg/day)	6.8 (4.5, 10.1)	4.0 (2.8, 5.3)	6.3 (4.8, 8.2)	8.1 (6.0, 10.7)	11.3 (8.1, 17.4)	< 0.001
Vitamin A (μg/day)	535.8 (337.5, 807.0)	285.5 (190.6, 422.6)	476.2 (346.5, 656.6)	641.2 (460.1, 879.5)	873.0 (600.6, 1210.2)	< 0.001
Zinc (mg/day)	12.6 (8.0, 20.8)	6.7 (5.0, 8.8)	10.6 (8.3, 15.3)	16.2 (11.4, 21.2)	24.7 (18.2, 33.1)	< 0.001
Selenium (μg/day)	114.0 (80.8, 156.2)	70.5 (54.5, 89.6)	106.3 (86.8, 127.8)	133.9 (105.6, 165.5)	177.4 (136.3, 228.4)	< 0.001
CERAD test	25.1 (6.4)	24.1 (6.4)	25.1 (6.4)	25.4 (6.6)	25.9 (6.3)	< 0.001
AFT	16.9 (5.5)	15.3 (5.4)	16.6 (5.3)	17.5 (5.5)	18.0 (5.4)	< 0.001
DSST	46.8 (17.0)	40.5 (16.7)	46.3 (17.8)	49.4 (16.2)	51.0 (15.2)	< 0.001

Continuous variables are expressed as mean (SD), or median (interquartile range [IQR]). Categorical variables are presented as frequencies (%). CDAI, composite dietary antioxidant index; BMI, body mass index; CERAD, the Consortium to Establish a Registry for Alzheimer’s Disease; AFT, Animal Fluency Test; DSST, Digit Symbol Substitution Test.

### 3.2 Association between CDAI and CFI

The univariate analysis showed that age, gender, race, education level, marital status, family income, drinking, hypertension, diabetes, stroke, depression, total intake of energy, protein, carbohydrate and fat were associated with CFI (Table 2).

**Table 2:** Association between covariates and CFI.

Variable	OR (95% CI)	*p*-value
Age group		
60–69 years	1 (Reference)	
70–79 years	1.67 (1.39 ~ 2)	<0.001
80+ years	3.07 (2.42 ~ 3.9)	<0.001
Gender		
Male	1 (Reference)	
Female	0.66 (0.56 ~ 0.77)	<0.001
Race		
Hispanic	1 (Reference)	
Non-Hispanic White	0.39 (0.31 ~ 0.49)	<0.001
Non-Hispanic Black	0.92 (0.72 ~ 1.19)	0.537
Asian and others	0.52 (0.37 ~ 0.73)	<0.001
Education level		
Less than High school	1 (Reference)	
High school	0.33 (0.25 ~ 0.42)	<0.001
More than High school	0.14 (0.11 ~ 0.18)	<0.001
Marital status		
Married or living with a partner	1 (Reference)	
Living alone	1.41 (1.2 ~ 1.66)	<0.001
Family income		
<1.3	1 (Reference)	
≥1.3	0.34 (0.28 ~ 0.4)	<0.001
BMI (kg/m^2^)	0.99 (0.98 ~ 1)	0.098
Smoking status		
Never	1 (Reference)	
Former	0.99 (0.83 ~ 1.17)	0.893
Current	1.22 (0.95 ~ 1.58)	0.123
Drinking		
No	1 (Reference)	
Yes	0.72 (0.61 ~ 0.86)	<0.001
Hypertension		
No	1 (Reference)	
Yes	1.35 (1.14 ~ 1.59)	<0.001
Diabetes		
No	1 (Reference)	
Yes	1.53 (1.27 ~ 1.85)	<0.001
Stroke		
No	1 (Reference)	
Yes	2.39 (1.72 ~ 3.31)	<0.001
Depression		
No	1 (Reference)	
Yes	2.09 (1.57 ~ 2.79)	<0.001
Total energy (kcal/day)*	0.96 (0.95 ~ 0.97)	<0.001
Total protein (g/day)**	0.92 (0.9 ~ 0.95)	<0.001
Total carbohydrate (g/day)**	0.98 (0.97 ~ 0.99)	<0.001
Total fat (g/day)**	0.9 (0.88 ~ 0.92)	<0.001

BMI, body mass index; OR, odds ratio; CI, confidence interval. *Total energy was entered as a continuous variable per 100 kcal/day increase. **Total protein, total carbohydrate and total fat were entered as continuous variables per 10 g/day increase.

In the fully adjusted model (Table 3, model 3), multivariate logistic regression showed that each 1-unit increment in CDAI score was associated with a 5% decrease in the risk of CFI (95% CI: 0.92 ~ 0.98, *p* = 0.004). When examining CDAI using quartiles, the fully adjusted model indicated that individuals in the second (Q2), third (Q3), and fourth (Q4) quartiles of CDAI had an adjusted OR of 0.81 (95% CI: 0.61 ~ 1.06, *p* = 0.125), 0.69 (95% CI: 0.51 ~ 0.92, *p* = 0.012), and 0.59 (95% CI: 0.43 ~ 0.82, *p* = 0.002), respectively, compared to those in the lowest quartile (Q1) of CDAI.

**Table 3:** Association between covariates and CFI.

Variable	Model 1		Model 2		Model 3	
	OR (95%CI)	*p*-value	OR (95%CI)	*p*-value	OR (95%CI)	*p*-value
CDAI	0.93 (0.9 ~ 0.96)	<0.001	0.95 (0.92 ~ 0.99)	0.006	0.95 (0.92 ~ 0.98)	0.004
Quartiles						
Q1	1 (Reference)		1 (Reference)		1 (Reference)	
Q2	0.74 (0.57 ~ 0.96)	0.024	0.81 (0.52 ~ 0.93)	0.133	0.81 (0.61 ~ 1.06)	0.125
Q3	0.62 (0.47 ~ 0.80)	<0.001	0.69 (0.44 ~ 0.83)	0.013	0.69 (0.51 ~ 0.92)	0.012
Q4	0.52 (0.39 ~ 0.68)	<0.001	0.60 (0.76 ~ 0.94)	0.002	0.59 (0.43 ~ 0.82)	0.002
Trend test		<0.001		0.002		0.001

Model 1 was adjusted for age, race, gender, education level, marital status and family income. Model 2 was adjusted for Model 1 + BMI, smoking status, drinking, total energy intake, total protein intake, total carbohydrate intake and total fat intake. Model 3 was adjusted for Model 2 + hypertension, diabetes, stroke and depression. CDAI, composite dietary antioxidant index; CFI, cognitive function impairment, NHANES, the National Health and Nutrition Examination Survey; Q, quartiles; OR, odds ratio; CI, confidence interval.

**Figure 2:**
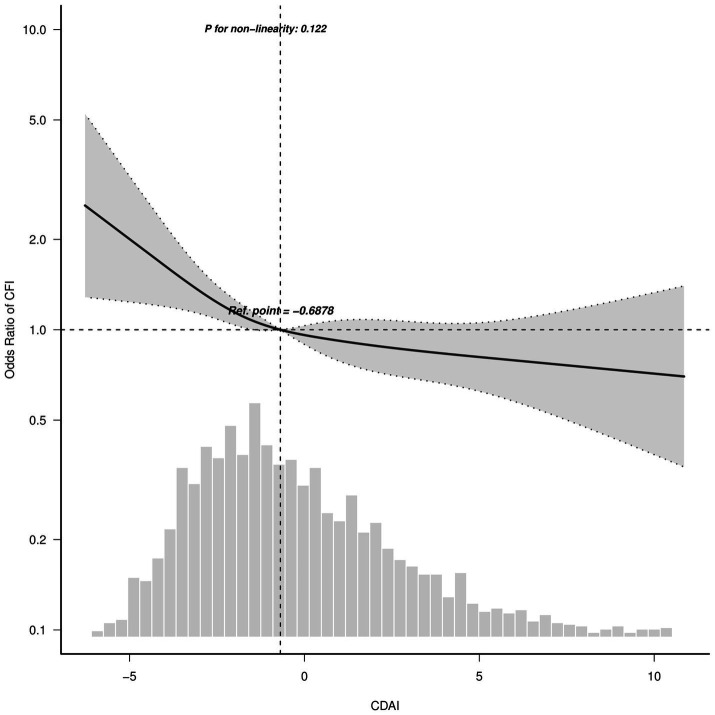
Association between CDAI and CFI odds ratio. Solid and dashed lines indicate the predicted value and 95% confidence interval. The restricted cubic spline model was adjusted for age, race, gender, education level, marital status and family income, body mass index, smoking status, drinking, total energy, total protein intake, total carbohydrate intake, total fat intake, hypertension, diabetes, stroke and depression. Ref., reference. Only 99% of the data is shown.

Restricted cubic spline regression was utilized to visualize the relationship between CDAI and CFI. With the increase in CDAI level, there was a noticeable decline in the risk of CFI, indicating a consistent inverse linear correlation between CDAI and CFI (Figure 2, *p* for non-linearity = 0.122).

Additionally, the relationship between CDAI and each component of cognitive function test was analyzed using linear regression. Except for CERAD test, the other two dimensions of cognitive function test were positively correlated with CDAI (Table 4).

**Table 4:** Linear regression model for CDAI on different test scores of cognitive function.

	CERAD test		AFT		DSST	
	β (95% CI)	*p*-value	β (95% CI)	*p*-value	β (95% CI)	*p*-value
CDCDAIAI	0.06 (−0.01 ~ 0.13)	0.093	0.08 (0.02 ~ 0.14)	0.008	0.18 (0.03 ~ 0.34)	0.022

The model was adjusted for age, race, gender, education level, marital status and family income, body mass index, smoking status, drinking, total energy, total protein intake, total carbohydrate intake, total fat intake, hypertension, diabetes, stroke and depression. CDAI, composite dietary antioxidant index; CERAD, the Consortium to Establish a Registry for Alzheimer’s Disease; AFT, Animal Fluency Test; DSST, Digit Symbol Substitution Test; CI, confidence interval.

### 3.3 Subgroup and sensitivity analyses

**Figure 3:**
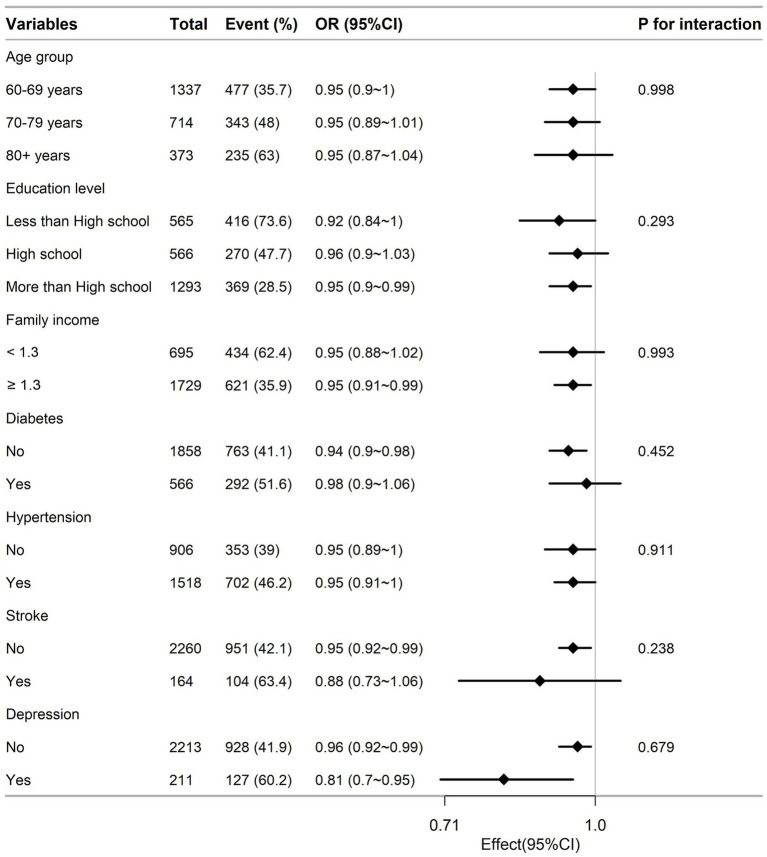
Association between CDAI and CFI according to general characteristics. Except for the stratification factor itself, the stratifications were adjusted for age, race, gender, education level, marital status and family income, body mass index, smoking status, drinking, total energy, total protein intake, total carbohydrate intake, total fat intake, hypertension, diabetes, stroke and depression.

Subgroup analyses were conducted to investigate the correlation between CDAI and CFI in various subgroups categorized by age, education level, family income, presence of diabetes, hypertension, stroke and depression. No significant interactions were found in any of the groups (Figure 3, *p* for interaction>0.05).

To ensure the stability of our findings, we further conducted sensitivity analysis by excluding individuals with extreme total energy consumption (total energy intake <500 kcal or > 5,000 kcal per day). The correlation between CDAI and CFI remained stable, as shown in Supplementary Table 1.

## 4 Discussion

In this study, we found that a greater CDAI, which suggests increased consumption of antioxidants in the diet, was associated with a lower risk of CFI in older adults residing in the United States. The association remains significant even after accounting for various demographic variables, lifestyle choices, and comorbidities. A dose–response analysis revealed a negative linear correlation between CDAI and CFI. Additionally, our findings were consistent across different clinical subgroups and in sensitivity analysis. These observations would have important implications for current CFI management strategies, especially for older Americans.

Many previous studies have investigated the relationship between different dietary antioxidants and cognitive function, but the research findings were inconsistent. A systematic review that included eight cross-sectional studies and 13 longitudinal studies found no evidence to support the use of vitamin C, vitamin E, and beta-carotene for preserving cognitive function (30). A randomized controlled trial (RCT) was conducted to determine if vitamin E or donepezil could delay the onset of AD in individuals with MCI. However, neither treatment showed any effectiveness in preventing the progression from MCI to AD (31). Similarly, a cognitive ancillary study conducted within the Women’s Antioxidant and Cardiovascular Study revealed that among women aged over 65 with pre-existing cardiovascular disease or risk factors for cardiovascular disease, supplementation with vitamin C, vitamin E, or beta-carotene did not slow down the rate of cognitive decline (32). A study conducted in France found that taking a combination of vitamin C, vitamin E, beta-carotene, zinc, and selenium daily significantly enhanced episodic memory and semantic fluency in individuals aged 45–60 years old (33). The inconsistent results may stem from various factors, including study design, dosages and types of antioxidants administered, cognitive tests utilized, and other relevant variables.

While it may be challenging to pinpoint the exact reasons for this discrepancy due to the heterogeneity among different studies, it is essential to consider that the majority of previous studies with negative results have primarily focused on single or fixed combination of antioxidants (31, 32), often overlooking the potential synergistic effects that may exist between different antioxidants. Assessing the combined effects of antioxidant on well-being could be more precise, given the possible overlap in food sources and protective functions for the brain across different antioxidants (34). According to Wright et al., CDAI has been linked to a decreased risk of lung cancer (11). Due to its convenient calculation, it has been considered as a practical tool for comprehensively evaluating dietary total antioxidant capacity (TAC). Several studies have demonstrated the correlation between CDAI and various illnesses including cancer (19), hypertension (12, 13), depression (16, 17) and so on. Although research on the relationship between CDAI and cognitive function is limited, our results align with the Singapore Chinese Health Study, which found a negative connection between increased TAC, as measured by both CDAI and Vitamin C Equivalent Antioxidant Capacity (VCEAC), and cognitive impairment (10). This convergence of results across different populations strengthens the evidence supporting the potential neuroprotective effects of multiple antioxidants.

Following the guideline of the Strengthening the Reporting of Observational Studies in Epidemiology (STROBE) statement, we conducted subgroup analysis to optimize data utilization and reveal the hidden reality. Upon conducting subgroup analysis, we found no notable interactions after stratifying the sample by key demographic and health-related variables, including age, education level, and presence of comorbidities. Incorporating sensitivity analysis enhanced the robustness of our research, demonstrating that the relationship between CDAI and CFI remained significant despite variations in overall energy intake. This suggests that our findings were not simply a byproduct of variable energy intake but may indeed reflect a link between diet and cognitive health. Cognitive function was assessed using a revised Mini-Mental State Examination (MMSE) in the Singapore Chinese Health Study, which may not provide a detailed assessment of specific cognitive domain deficits (10). In light of the three dimensions covered by the cognitive tests administered in NHANES, we conducted additional analyses to investigate how CDAI is related to particular cognitive functions. Our results indicated a linear correlation between CDAI and both DSST and AST, while no significant correlation was observed with CERAD test. These findings suggested that antioxidants might have a more pronounced protective effect on executive function, attention and processing speed, as assessed by AFT and DSST, respectively. Additional studies are required to ascertain if antioxidants provide advantages to a particular cognitive field.

The CDAI encompasses six antioxidants, including vitamins and micronutrients. Despite the mechanisms behind the relationship between CDAI and CFI still needing further investigation, several neuroprotective mechanisms of antioxidants from the diet have been elucidated. Firstly, vitamins with antioxidant properties can directly neutralize oxygen free radicals. For example, vitamin E exerts its antioxidant effect by protecting cellular membranes and neutralizing oxygen free radicals generated by polyunsaturated fatty acids (35). Acting as a direct scavenger, it directly neutralizes superoxide and hydroxyl radicals, providing additional protection against oxidative damage to cells (35). Treatment of vitamin E significantly increased the neuronal survival by inhibiting ROS formation and lipid peroxidation in a kainic acid-induced neuronal death model (36). Secondly, certain antioxidants have been shown to promote mitochondrial function. In a rat model of AD, co-administration of zinc and selenium resulted in notable improvements, which included reduced mitochondrial dysfunction, decreased levels of ROS and lipid peroxidation, as well as enhanced cognitive performance. Simultaneously, there was a notable rise in the functioning of key antioxidant enzymes such as superoxide dismutase, glutathione peroxidase, and catalase within the brain’s mitochondria (37). Moreover, antioxidants may play a role in regulating synaptic plasticity. The decline in synaptic plasticity in the hippocampus has been considered as a crucial factor in the deterioration of cognitive function during aging (38). Animal models of vitamin A deficiency displayed brain alterations resembling those observed in aging, such as a decrease in hippocampal volume, impaired synaptic plasticity, and reduced neurogenesis, all of which could be reversed by administering retinoic acid (39).

Our study has some strengths. Firstly, the utilization of a nationally representative sample enhances the applicability of our findings. Secondly, we employed CDAI to accurately and consistently measure antioxidant capacity from diet. Additionally, our study considered both dietary and supplemental sources of antioxidants, providing a comprehensive understanding of overall antioxidant intake. Lastly, the comprehensive nature of the NHANES dietary data collection further strengthens the robustness of our findings.

However, it is important to acknowledge the constraints of our research. The cross-sectional design restricts the ability to infer causation. Long-term cohort studies are necessary to validate the directionality and durability of the identified connections. The dietary recall method, despite its thoroughness, is susceptible to recall bias and may not capture long-term dietary patterns. Furthermore, CDAI only calculates the intake of six specific antioxidants available in the NHANES database, which may not fully reflect the overall antioxidant capacity. It is also unable to estimate the bioavailability and bioactivity of antioxidants. Additional large randomized controlled trials or observational studies are necessary to further explore the relationship between antioxidants and cognitive outcomes.

## 5 Conclusion

In conclusion, this cross-sectional study based on two cycles (2011–2014) of data from the NHANES database demonstrated an inverse linear relationship between CDAI and CFI among older adults in the United States. Even after accounting for demographic, lifestyle, and health-related covariates, the results continued to show statistical significance, indicating that higher levels of CDAI are associated with a lower risk of cognitive decline in a clear dose-dependent pattern. Future studies, including randomized controlled trials and longitudinal cohort studies, are indispensable to validate these findings and explore the mechanisms by which dietary antioxidants may influence cognitive outcomes.

## Data Availability

The datasets presented in this study can be found in online repositories. The names of the repository/repositories and accession number(s) can be found at: https://wwwn.cdc.gov/nchs/nhanes/Default.aspx.

## Footnotes

^1^ http://www.R-project.org

^2^ http://www.clinicalscientists.cn/freestatistics
