# Supplementary material for: Association between composite dietary antioxidant index and cognitive function impairment among the US older adults: a cross-sectional study based on the NHANES 2011–2014
Source: Front Nutr. 2024 Nov 22;11:1471981. doi: 10.3389/fnut.2024.1471981 (PMC11622812; doi:10.3389/fnut.2024.1471981)
Supplement: Supplementary file 1 [file Table_1.DOCX]

**Supplementary material Table 1** Sensitivity analysis (Excluding total energy intake <500 kcal or >5000 kcal).

| Variable | OR (95%CI) | *p*-value |
| --- | --- | --- |
| CDAI | 0.95 (0.92~0.98) | 0.005 |
| Quartiles |  |  |
| Q1 | 1 (Reference) |  |
| Q2 | 0.82 (0.62~1.08) | 0.158 |
| Q3 | 0.70 (0.52~0.94) | 0.017 |
| Q4 | 0.60 (0.43~0.83) | 0.002 |
| Trend test |  | 0.002 |

CDAI, composite dietary antioxidant index; CI, confidence interval; OR, odds ratio.
